# AGENT ORANGE‐INDUCED Anaplastic Large‐Cell Lymphoma (ALCL) with Cutaneous Involvement

**DOI:** 10.1002/ccr3.4041

**Published:** 2021-03-11

**Authors:** Morgan Lorio, Brandon Lewis, John Hoy, Matthew Yeager

**Affiliations:** ^1^ Advanced Orthopedics Altamonte Springs FL USA; ^2^ East Tennessee University College of Nursing Johnson City TN USA; ^3^ Chicago Medical School at Rosalind Franklin University North Chicago IL USA; ^4^ Herbert Wertheim College of Medicine at Florida International University Miami FL USA

**Keywords:** Dermatology, Oncology, Orthopaedics

## Abstract

Anaplastic large‐cell lymphoma (ALCL) is a CD30 + lymphoproliferative disorder that may manifest with skin involvement.^1^ We present a rare case of Agent Orange‐induced ALCL with cutaneous involvement of the hand, surgical excision, and follow‐up treatment.

## Graphical abstract

We present a case of Agent Orange‐induced ALCL with cutaneous involvement of the hand and the subsequent surgical interventions. This case underscores the importance of early referral of hand masses to a hand surgeon specialist.

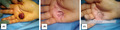

## 1 INTRODUCTION

Anaplastic large‐cell lymphoma (ALCL) is a CD30 + lymphoproliferative disorder that can involve the skin. The characteristics of primary and secondary ALCL include raised red skin lesions, nodules, or tumors.^1^ More than 3 million Americans served in the Vietnam War. Some of them were exposed to defoliant chemicals, including Agent Orange. Lasting health effects are known to be caused by these exposures, including types of cancer. This case study presents a bizarre case of Agent Orange‐induced ALCL and reviews the presentation, surgical excision, and follow‐up treatment that was involved.^2^

## 2 CASE PRESENTATION

A 63‐year‐old right‐hand dominant man initially presented in 2010 to his primary care physician (PCP) with a one‐centimeter erythematous raised lesion on his right palm. He reported that he was in his usual health until three weeks ago when he noticed a point of irritation on the palmar aspect of his right hand. It began to form into a mass with increased sensitivity. It had a rapid onset from the time it was first apparent as it increased to the size of a silver dollar with increasing sensitivity over the course of three weeks, according to the patient.

On presentation, there was no preceding trauma to the area—although the patient worked with his hands and acknowledged the possibility for an implanted foreign body that he could not specifically recall. His past medical history is significant for hypertension and hyperlipidemia with a myocardial infarction in 2007; however, this did not require bypass or stenting. Additionally, he presented with a history of gastroesophageal reflux disease, benign prostate hypertrophy, and noninsulin‐dependent diabetes mellitus. The patient's family history was notable only for one case of lung cancer. He is married and a Vietnam War veteran where his service duties included preparing aircraft for flight. He is a previous smoker, having quit three years ago after a 30 pack‐year smoking history. He does not use alcohol, illicit substances, or over‐the‐counter supplements. His medications at that time were as follows: Latanoprost 2.5ml both eyes, Tamsulosin 0.4mg QHS, pravastatin 40mg, metoprolol 25mg BID, acetylsalicylic acid 81mg, Omeprazole 20mg, and metformin 500mg daily. He reported allergy to Avodart and Simvastatin.

At that time, his vital signs were normal, and physical examination showed a lesion on the volar aspect of the right hand. The lesion was approximately 1.5‐2.0 cm across and tender to palpation. The mass was aspirated, and the specimen sent for culture. At this point, the presumptive diagnosis was made of foreign body. The patient was discharged on ten days of Keflex with an appointment to see his PCP at the Veterans Affairs (VA) clinic in two weeks.

Eleven days later, the patient presented again at the same facility through the emergency department with a complaint of escalating symptoms relating to the right upper extremity. His pain had increased since the previous visit, and the appearance of the mass had progressed. On examination, the lesion on the right hand had become a raised erythematous mass that is centrally excoriated and 2.0 cm in diameter. Further, there was apparent lymphangitis from the lesion not surpassing the antecubital fossa. The anterior aspect of the forearm was found to be warm and tender, corresponding with the area of erythema.

An ultrasound study of the right upper extremity was ordered to investigate the possibility of a deep venous thrombosis (DVT), and orthopedics was consulted. The ultrasound study found no evidence of DVT. Orthopedics noted that the mass appeared to be consistent with squamous cell carcinoma and recommended the patient see a hand surgeon with specific concerns for closure postresection of the lesion. The patient was referred to a hand surgeon at the VA as the patient was anxious to leave and return home. With knowledge of his upcoming appointments and with instruction to return if symptoms progressed, the decision was made to discharge him on clindamycin 300 mg TID and Relafen 500 mg BID.

**FIGURE 1:**
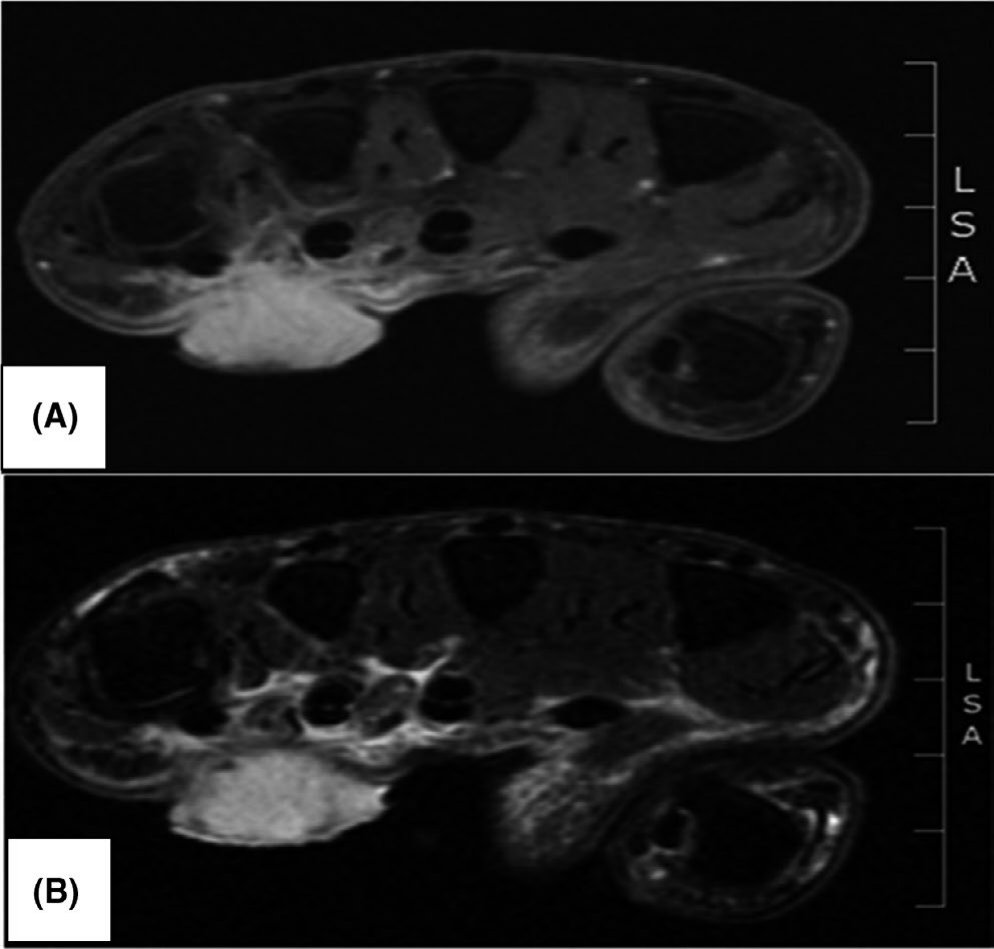
A, Axial T1 fat‐suppressed postcontrast image demonstrates a superficial polypoid avidly enhancing lesion measuring 1.7cm AP x 2.1cm TV x 2.0 cm SI and located on the volar side of the right hand between the distal 4th and 5th metacarpals; the lesion infiltrates the palmar aponeurosis and extends within the central compartment; there is an enhancement of the third and fourth lumbrical muscles and lesion extension up to the flexor digitorum superficialis and profundus IV and V; B, Axial T2 fat‐suppressed image shows T2 hyperintensity within the superficial lesion; mild amount of edema seen within the central compartment

The patient next initiated contact with our office with the hope of a more rapid resolution of intractable pain. Upon the initial visit to our hand surgery clinic, the patient's right upper extremity was found to have a lesion on the volar aspect of the hand. Imaging of the hand was done to delineate the extent of the mass with the intent of excisional biopsy. MRI with gadolinium showed a “mass involving the palmar aspect of the hand at the level of the fourth and fifth rays. This mass superficially is relatively circumscribed but does extend into the deeper soft tissues where it is more ill‐defined.” It was seen to be hypertrophic at the base with induration at its central portion (see Figure 1A and 1B). The lesion presented as an ulcerated, erythematous, granulating tissue nodule, projecting from the plane of the palm to a height of approximately half its diameter. The lesion was found to be tender in the immediate area, and the presentation was devoid of other positive findings including B symptoms. The lymph node examination was negative at the ipsilateral epitrochlear, axillary, and supraclavicular levels. The patient was found to have a white count of 10.1 with an elevated neutrophil fraction of 80% and an erythrocyte sedimentation rate (ESR) of 28.

**FIGURE 2:**
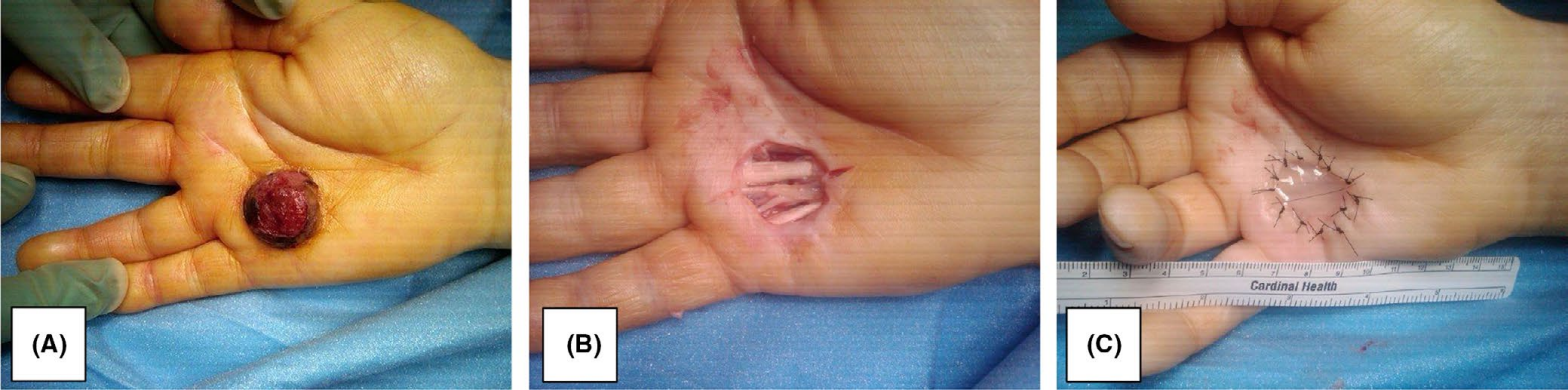
A, Preoperative mass; B, intraoperative view with mass excised; C, postoperative closure with Integra

The patient was scheduled for surgical resection six days following the visit. The patient underwent excisional biopsy of the mass (see Figure 2A) under a tourniquet, with the dissection going down to the flexor sheath underlying the mass lesion (see Figure 2B). A margin of 1‐3 mm was left between the mass and the line of dissection, and afterward, the surgical edge was marked on the specimen and sent for pathology. The wound extended from the lateral portion of the fifth ray to the midline of the middle finger. The flexor sheath was then excised after an assessment of its involvement produced fluid abnormal for the anatomy. This fluid was collected and sent to pathology. The wound was provisionally closed with Integra bilaminar xenograft^3^ (see Figure 2C) as a bridge, given the size of the resulting defect and the potential for further procedures pending pathological analysis, thus avoiding flap morbidity.

The specimen was sent to pathology for tissue analysis, Gram stain, and culture including fungus, acid‐fast bacillus (AFB), tuberculosis (TB), and mycobacterium. Intraoperative Gram stain revealed gram‐positive cocci in pairs. Consultation was made with infectious disease who recommended Vancomycin and Rocephin empirically, pending culture results.

Although the postoperative Technitium 99 bone scan showed small increased activity on the left anterior aspect of the L5 vertebral body and decreased uptake of the nucleotide within the distal femur, a negative study was, overall, concluded. Cultures grew a gram‐negative rod (Escherichia coli) with sensitivity studies revealing Ciprofloxacin to be the agent of choice. Thus, the patient's antibiotics were changed accordingly. CT scan of the chest showed an ill‐defined mass, 46 mm diameter, in the right axilla as well as several smaller masses consistent with lymph nodes in the area. Three noncalcified nodules were seen in the lungs of 3‐4 mm diameter each, without the option for biopsy: “Their small size also precludes a percutaneous biopsy.”

**FIGURE 3:**
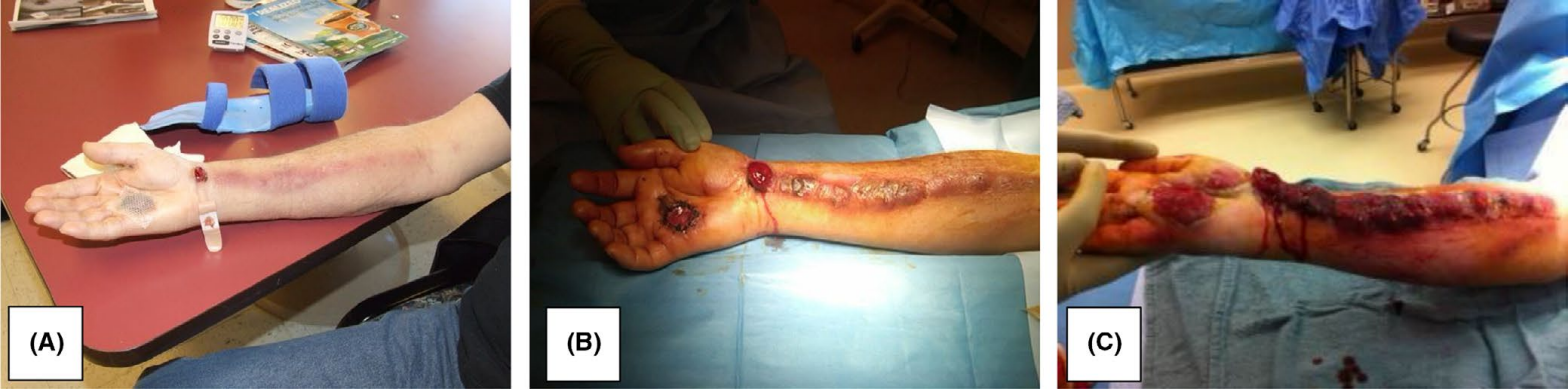
Ascending malignancy: A, 8 days postexcisional biopsy with proximal erythema and new mass; B, intraoperative view: subsequent incisional biopsy; C, intraoperative photograph of ELD from receiving tertiary care center

Eight days later, the patient returned to the clinic with increased involvement of the right forearm (see Figure 3A). His right upper extremity was found to be streaked with erythema on the anterior aspect. The area was found to be warm and tender on examination. An additional lesion had formed over the volar aspect of the right wrist, and new lymphadenopathy was found in the medial aspect of the right axilla. The patient continued to deny B symptoms, no fever, chills, weight loss, or fatigue. He reaffirmed no previous similar lesions. Antibiotics were extended; an MRI of the right axillary region was ordered to assess the extent of the involvement, and an appointment was made for follow‐up in one week. MRI revealed contrast‐enhancing right axillary soft tissue masses—the largest of which was measured to be 72 x 73 x 67 mm as well as several enlarged lymph nodes. In comparison with the CT scan of the chest several days earlier, the largest mass was found to have increased in size within the time period. The clinical presentation in conjunction with imaging findings increased concern for metastatic adenopathy, as commented on by the radiologist: “This is most likely due to metastatic disease with low probability of reactive adenopathy from an infectious etiology.” However, they continued to note that “Lymphoma is also a possibility although for lymphoma to present as a peripheral distal extremity mass would be unusual.”

Over the next couple of days, the patient's symptoms mimicked that of suppurative thrombophlebitis of the forearm, which progressively consolidated and visually proliferated and/or metastasized via the lymphatic tracts to the right axilla. Overnight, the ascending malignant oddity sprouted through the flesh causing a bizarre disruption of the soft tissue integument (see Figure 3B). At the request of pathology, another biopsy (incisional as opposed to excisional) was obtained for examination via flow cytometry at the Mayo. At this time, the family of the patient revealed he had been responsible for transporting and loading Agent Orange during his deployment in the Vietnam War. Transfer to Vanderbilt (tertiary care center) was initiated to assist in anticipated complications with extensive limb disease (ELD) management (see Figure 3C).

**FIGURE 4:**
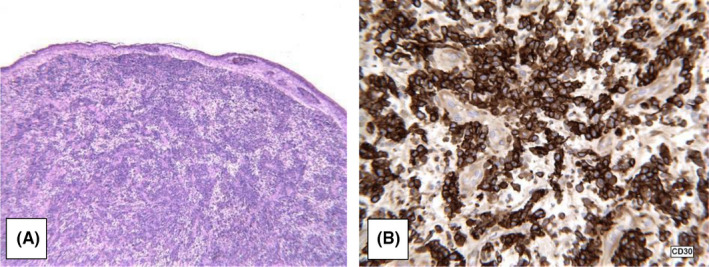
A, Low power H&E; B, CD30 expression

Twenty‐two days later, the pathological analysis returned from this institution reporting:

“This was a difficult case because the histological features don’t resemble those typically associated with a lymphoproliferative disorder. On histology, the lesion consists of sections of skin, with a suggestion of a collarette and overlying ulceration, a pattern that can be seen in a pyogenic granuloma … Indeed there is prominent vascularity present with the large malignant cells present in the interstitium, some of these are spindled. Mitotic activity is present with abnormal forms and nuclei that are large and vesicular.” (See Figure 4A and Figure 4B).

Thus, the preliminary diagnosis was determined to be malignant neoplasm, NOS. The tissue was then subjected to a battery of immunoperoxidase stains, verified and extended at an outside institution. The specimen initially tested positive for epithelial membrane antigen and Vimentin and negative for CD68, S100, pancytokeratin, CD34, Melan‐A, HMB‐45, desmin, actin, Bartonella henselae, CK7, CK20, CK5/6, and P63. Follow‐up testing showed the specimen was positive for CD2 and CD4 but was lacking other T‐cell associated antigens, CD3, CD5, and CD7. Further tests were run for leukocyte common antigen (CD45), CD20, CD3, CD30, broad‐spectrum keratin, ALK protein, and CD31. The neoplastic cells were found to be strongly positive for CD45 and CD30. They were negative for all other markers. Flow cytometry confirmed CD45 positive and provided evidence of an aberrant T‐cell phenotype with the positivity of CD2 and CD4 and negativity for other T‐cell antigens. The outcome found the specimen to be CD30 and CD45 positive and ALK negative—likely CD30 lymphoproliferative neoplasm and was subsequently sent for fluorescence in situ hybridization (FISH) studies.

The final diagnosis was deduced from the pathology, which was found to be CD30+, ALK‐, T‐cell non‐Hodgkin, and anaplastic large‐cell lymphoma (ALCL) involving the skin. This diagnosis and case presentation were accepted by the US Department of Veterans Affairs as an Agent Orange‐related lymphoproliferative disorder. Due to lack of widely available PET‐CT at the time, it is not possible to completely rule out systemic anaplastic large‐cell lymphoma with cutaneous involvement in favor of the diagnosis of PC‐ALCL. Due to the ascending metastasis, timeline of appearance of axillary adenopathy, and pathology analysis, it was surmised that the lesion began as a primary cutaneous lesion in the hand and transmogrified at an accelerated rate to its current form. The patient was initiated into CHOP (cyclophosphamide, hydroxydaunorubicin, vincristine, prednisolone), ICE (ifosfamide, carboplatin, etoposide), and methotrexate‐based chemotherapy for a five‐month period in addition to radiation. The skin lesions resolved on gross inspection consistent with clinical complete response (CCR) in subsequent hand clinic follow‐up. However, unfortunately, he passed away 12 months following his primary surgical excision due to relapse defined as extracutaneous pulmonary recurrence after CCR.

## 3 DIFFERENTIAL DIAGNOSIS

In the course of this case, there was significant time between examining the patient for the first time in the hand clinic and when the pathology gave a definitive diagnosis. From our initial visit, the differential considered for an eroded mass lesion on the palmar aspect of the right hand initially included foreign body, squamous cell carcinoma, pyogenic granuloma, and/or lymphoma (see Table 1). These were consistent with both the history and the physical examination and are some of the more common diagnoses in a longer list considered.

**TABLE 1:** Presentation, diagnosis, and treatment. Comparing embedded foreign body, squamous cell carcinoma, pyogenic granuloma, and PC‐ALCL

*Pathologies Considered: Classic Presentation, Diagnosis, and Treatment*		
	*Presentation*	
	*Diagnosis*	*Treatment*
Foreign Body	Raised erythematous area, tender to palpation, with systemic symptoms of fever, elevated WBC	
	Imaging (x‐ray), direct visualization of, or history consistent with embedded foreign body	Therapeutic excision of foreign body, antibiotics
Squamous Cell Carcinoma	Presents in all ages, but more commonly older populations in sun‐exposed areas. Appears as scaly red patches, open sores, wart‐like skin, or rapid growth with central depression. May crust over, itch or bleed.	
	Characterized by abnormal, accelerated growth of squamous cells	Treatment varies, may require surgery, chemotherapy, and radiation
Pyogenic Granuloma	Polypoid form of capillary hemangioma Rapidly growing exophytic red nodule with a collar of hyperplastic epidermis at the base. Bleeds easily and is often ulcerated or crusted. Tender to palpation. Often appears on the fingers, lips, mouth, trunk, and toes. 1/3 are trauma‐related, growing to 20 mm in a few weeks	
	Excisional biopsy Histology shows exuberant granulation tissue, with edema and numerous neutrophils.	Surgical biopsy (excision)
Primary Cutaneous Anaplastic Large‐Cell Lymphoma	Presents at a median age of 60 years old; aggressive often with lymph node and soft tissue disease. Solitary reddish to brownish nodules and tumors, which frequently ulcerate. Possible to see multiple in clusters. Tender to palpation, B symptoms	
	Biopsy with flow cytometry demonstrating Cytotoxic T‐cell origin CD4 + and CD30+, sometimes with ALK rearrangements (2p23). Hallmark cells: large anaplastic cells with horseshoe‐shaped nuclei and large cytoplasm.	Excision of mass followed by chemotherapy (CHOP, ICE, methotrexate), consider radiation therapy Associated with favorable prognosis and disease‐related 5‐year survival rate of 90%‐95%

### 3.1 Foreign body

Alternatively, the patient was employed in a manual labor trade and indicated during the interview that he had a history of embedded foreign bodies as a result of occupational hazard; thus, foreign body was included in the differential. It has been postulated that embedded foreign bodies are the most common hand injury, estimated at 41% in one review—many of which happen on the job.^4^ More often than not, fragments are extricated on‐site by the injured party. This significantly reduces the number of visits to a physician for removal and thus decreases its prevalence deduced from the study in the medical setting.

Lacerations may often provide an entry point for a foreign body; a fragment of the cutting implement or materials between the skin and the implement may become lodged in the wound. Lacerations account for more than 4 million ED visits annually^5^ and just over 2 million on the upper extremity accounting for 1.9% of all patients to the ER.^6^ Many of these lacerations are complicated by the presence of a foreign body. One review found that it may be missed on initial examination up to 38% of cases.^4^ A review of 200 cases found males are at increased risk (2.3:1) with 37.5% in the hand or wrist and 40.5% work‐related. The average time of removal of the foreign body was 7 months with 43% removed within a week and 11% remaining embedded for over a year.^3^ In some cases, the initial traumatic event may not be recalled by the patient (up to 62% in one case series). Also, it is often associated with manual laborers who suffer multiple minor traumas to the hands regularly. A retained foreign body puts the patient at risk for delayed wound healing, infection, allergic reaction, tissue damage, granulomas, pyogenic granulomas, pain, and disability.^4^, ^7^

Patients should be evaluated with a thorough history and physical examination, including vascular, motor, and sensory examinations and two‐handed deep palpation over the area, exploring for sharp pain with deep palpation or pain associated with a mass. Lacerations should be thoroughly explored under bright light with margins extended as needed for direct visualization. Roentgenograms are helpful for metal and most glass; however, they have poor sensitivity for organic material—in particular, wood splinters. In one review, 88% of the false negatives on roentgenogram involved wood, while another study found that only 15% of known embedded wooden foreign bodies were able to be identified. While the wood itself is not visible, secondary changes (such as lytic lesions, periosteal reaction, and pseudotumors of soft tissue) may indicate such an organic foreign body.^4^, ^7^ Ultrasound, fluoroscopy, and computed tomography (CT) may also be used to aid in detection and localization. Other indicators of a retained foreign body are relapsing infection or patient perception.

Once the diagnosis has been confirmed, a decision must be made with regard to removal. Foreign bodies that are biologically reactive or are near sensitive structures, such as vessels or tendons, should be removed unless anatomy prevents access. Although if the plan tends toward leaving the material in situ, the prospect of damage to adjacent anatomic structures must be considered as objects may migrate within the soft tissue over the course of time.^8^ Removal is the preferred method of treatment in general and should be carried out in a setting that provides adequate anesthesia and equipment for the surgeon. Bright light and magnification are recommended; tourniquet and fluoroscopy may also be beneficial in some cases. Surgical specimens should be sent for aerobic and anaerobic culture as well as histology if there are tissue changes of concern. While this patient had failed antibiotic treatment, the possibility remained that the antibiotic coverage was inadequate or that he would benefit from the removal of the embedded object.

### 3.2 Squamous cell carcinoma (SCC)

Squamous cell carcinoma (SCC) was also included as a possible diagnosis. As early as 1775, Percivall Pott drew the correlation between cancer of the scrotum and exposure to soot in chimney sweeps. He was followed by PG Unna in 1894 who observed that extensive exposure to sunlight may be related to tumors in the skin.^9^

Today, it is recognized that there are many risk factors for the development of SCC, including polycyclic aromatic hydrocarbons and sunlight. As a group, nonmelanoma skin cancer is now the most common cancer in the United States with over 1.3 million cases in 2001.^10^ Approximately 20% of these are SCC, making it the second most common nonmelanoma skin cancer and the second most common cancer among the white population.^11^, ^12^ While comprising 4% of all malignancies in the United States,^13^ SCC is unlike basal cell carcinoma (BCC) in that it is associated with a substantial risk of metastasis and a regional distribution based on the site of origin. SCC often arises in sun‐exposed skin. Most are identified in the physician's office, and smaller lesions are removed in the office as a minor surgical procedure. Larger and more invasive lesions require more aggressive surgical management, radiation therapy, or both. The primary risk factor for SCC is cumulative lifetime sun exposure—in particular, the sunlight in the UVB spectrum (290‐320 nm). This has been well illustrated in population studies based on latitude which found that there is a linear increase in prevalence with proximity to the equator corresponding to incident solar UV radiation.

In addition, SCC patients usually present in patients over 70 years and maintain a 3:1 male to female ratio. There is also a 2.5x risk increase for those who utilize tanning devices.^14^ Persons with a fair complexion, albinism, blue or gray eyes, blond or red hair, those who burn easily when exposed to sunlight, or have a history of sunburns are similarly at higher risk for SCC. Those with Fitzpatrick skin type I or II account for most cases of SCC, and there is up to a fivefold increase in risk for those with occupational exposure to ultraviolet light.^15^, ^16^ Additionally, xeroderma pigmentosum, albinism, and immune systems suppression increase the risk of a primary lesion and, furthermore, often develop multiple SCCs. Ionizing radiation, PUVA, and some infections (including HPV 6, 11, and 16) and exposure to arsenic have been shown to increase the risk of SCC. Associations have also been found in populations with a history of alcohol or tobacco use or a history of previous nonmelanoma skin cancer.

Tumors with a diameter of less than 20 mm are associated with a 9.1% rate of metastasis and a 5.8% rate of recurrence versus those greater than 20 mm for which metastasis and recurrence rates are 15.7% and 23.4%, respectively. Lesions deeper than 4 mm are associated with a metastatic rate of up to 30.3%, and all lesions resulting in death were deeper than 10 mm.^10^, ^17^ While most are found on the head and neck (51.1%), 20.5% of the lesions are found on the upper extremities.^14^ Frequently, the SCC lesion is preceded by actinic keratosis. The lesion itself commonly appears as a firm, smooth papule or plaque, which often have heaped‐up edges. It may present as a pink nodule with or without overlying surface changes including scaling or ulceration. Patients report that the lesion bleeds with minimal trauma. In addition to being grossly characterized (ie, diameter, depth, rate of growth), lesions need to be assessed for lymphatic spread and staged using the TNM system.

Management ranges from topical therapies (chemotherapy, radiation therapy, cryotherapy) to surgical (electrodessication and curettage, excision, laser surgery, Mohs surgery), or systemic (chemotherapy). Histologic confirmation of the lesion is preferred. It is often completed prior to definitive treatment. Verification of clear margins is preferred, especially in larger or more aggressive cases.

This patient's lesion was ulcerated with a border consistent with SCC. He had sustained significant UVB exposure on his hands during his time in the military serving in Vietnam in addition to handling caustic, bioactive chemicals. In fact, he had increased UV exposure all his life having lived in the southern United States since he was a child.

### 3.3 Pyogenic granuloma

The presentation appeared to be consistent with a pyogenic granuloma (lobular capillary hemangioma), a relatively common benign vascular proliferation lesion in the surface of the cutaneous or mucous membrane. These comprise 0.5% of all skin nodules with an average lesion diameter of 6.5 mm with an epithelial collarette at the base.^18^, ^19^ These lesions are misleadingly named, being that they are neither infectious nor granuloma forming. They have been found to appear in children and young adults following physical trauma (in 7% of cases)—although it is probable that the majority arise de novo. In adult patients, the average age at treatment is around 47 years (patients < 18 years excluded) with a 1:1 ratio for cutaneous lesions.^20^ They usually present as a solitary erythematous (red to purple) sessile or pedunculated mass, which may be ulcerated and prone to bleeding.^18^ They develop in a subacute time frame, most commonly on the trunk and upper extremities (32% and 28% of cutaneous lesions).^19^ They also may appear in mucosal tissue (12% of PG lesions), which are often associated with pregnancy. Up to 5% of second‐ or third‐trimester gravida may present with these. Most appear on oral mucosa under what is presumed to be hormonal influences.^21^ Other known associations are Bartonella species seropositivity.^22^ While those left untreated may eventually atrophy and slowly regress, removal is indicated for those that cause the patient discomfort, incessantly bleed, or are of cosmetic concern. Full‐thickness excision has the lowest rate of recurrence (2.9%‐3.7%) in comparison with other options^18^, ^19^, ^20^, ^23^ while allowing for histopathologic confirmation of the diagnosis. Lesions in areas in which residual scar would be deemed cosmetically unacceptable may be treated using liquid nitrogen cryotherapy, which often leaves less scarring than surgical removal and provides a low recurrence rate (as low as 1.6%)^21^; however, it may require more than one treatment and does not allow for histopathologic confirmation. This may be a problem as these are misdiagnosed with some frequency, up to 18% in one study.^24^

### 3.4 Primary cutaneous anaplastic large‐cell lymphoma (ALCL)

Primary cutaneous anaplastic large‐cell lymphoma (ALCL) is a T‐cell proliferation that presents in the skin without systemic involvement. These are CD30 + ALCLs which do not have the anaplastic lymphoma kinase (ALK) translocation typical of systemic ALCLs (t(2;5), creating NPM‐ALK). Diagnostic evaluation should include a detailed history and physical examination with close attention to the report of B symptoms, lymph node involvement, or other indications of extracutaneous involvement.

Clinical manifestations determine whether the lesion falls into one of two groups: (1) primary cutaneous without extracutaneous involvement at presentation or (2) systemic form with secondary skin involvement. The primary cutaneous form generally has a better prognosis as the neoplasm tends to be indolent and may even spontaneously regress in up to 25% of cases. Histologically, ALCL is characterized by sheets of large CD30‐positive tumor cells. These cells are characteristic of anaplastic cells with irregularly shaped nuclei, eosinophilic nucleoli, and abundant cytoplasm. Less commonly, they demonstrate Reed‐Sternberg–like cell morphology with a high mitotic index.^1^ Immunohistochemistry demonstrates activated CD4 + T cells with variable loss of CD2, CD3, or CD5 antigens. CD30 is typically expressed by the majority of the neoplastic cells. Cutaneous lymphocyte antigen (CLA) expression is positive unlike the systemic CD30‐positive lymphomas; however, epithelial membrane antigen (EMA) and the ALK translocation are not seen in the primary cutaneous though often observed in the primary systemic form. ALK‐negative systemic ALCLs tend to be more aggressive and are more likely to relapse than ALK‐positive subtypes—although this aggressive nature in the absence of ALK does not hold for PC‐ALCL. While ALK‐positive (systemic) forms of cancer proliferate as a result of constitutive phosphorylation due to the ALK promotion, the driving force behind the ALK‐negative primary cutaneous versions are not well understood. Primary cutaneous ALCL tends to favor the older patient (median age, 61 years) with a male predominance. It usually presents as a single or localized cluster of erythematous skin nodules which often ulcerate.

Following a thorough history and physical examination, these patients should be assessed for systemic involvement through imaging, utilizing a chest x‐ray or CT scan. MRI can be helpful to assess bone marrow involvement and bone scanning, or PET scanning may be used in conjunction. A biopsy is necessary to confirm diagnosis and excision with or without adjuvant radiotherapy may be considered for localized skin lesions. Multidrug chemotherapy (eg, CHOP) may be considered in patients with systemic disease. While the ALK‐negative systemic form has a bleak outlook (5‐year survival rate of 36%‐37%^25^, ^26^), generally, cutaneous CD30 + anaplastic large‐cell lymphoma has a favorable course with a 90% disease‐related survival rate at 5 years.^27^

In this particular case, the patient's history of exposure to Agent Orange necessitated follow‐up with the appropriate organizations. Veterans and Agent Orange (VAO) is the committee assembled to review the health effects of exposure to herbicides in Vietnam Veterans. Non‐Hodgkin lymphoma is one of a handful of “health outcomes” recognized by the VAO as having sufficient evidence of an association with prior herbicide exposure.^28^

## 4 DISCUSSION

We present a unique case demonstrating the dilemma of diagnosing and managing a hand lesion of a rather bizarre nature. The characteristics associated with this particular lesion presented a problematic situation at best. We performed an excisional biopsy, which appeared to be in line with the potential diagnoses of an embedded foreign body which has failed antibiotic treatment, squamous cell carcinoma, pyogenic granuloma, or cutaneous anaplastic large‐cell lymphoma (ALCL). This step helped to treat the lesion while providing a biopsy for further testing. Additionally, the operative histology excluded squamous cell carcinoma (SCC). This would then be followed by a pathologic evaluation to confirm the diagnosis and direct further treatment. In the case of ALCL, subsequent therapy would be necessary in the form of radiation or chemotherapy, in contrast with no additional management in the case of pyogenic granuloma.^23^, ^29^ A foreign body once removed may be covered with a broad spectrum of antibiotics as dictated by clinical indicators. In retrospect, our approach is still in line with the current recommendation for treatment of PC‐ALCL, the diagnosis made at the time, which is complete surgical excision and/or local radiotherapy.^30^, ^31^

Non‐Hodgkin lymphoma is a particularly rare occurrence on the hand, and its rarity contributes to issues in prompt and proper diagnosis. It is difficult for a clinician to accurately determine the etiology of certain lesions without the aid of flow cytometry or FISH studies, thus warranting invasive biopsy for pathologic diagnostic procedures. Clinicians require the knowledge of an exact diagnosis to assess morbidity and mortality and thus accurately elucidate an appropriate treatment plan.

Unfortunately, diagnostic pathology analysis for complex cases such as this can take upwards of 7‐14 days, or more in some circumstances, to establish a sound diagnosis in order to direct further surgical procedures, chemotherapy, radiation, or some combination treatment. This dilemma can provide both the clinician and the patient with less than desired treatment outcome. To our knowledge, no viable solution to optimize the outcome of this dilemma is currently available; however, the rarity of this Agent Orange‐induced ALCL with cutaneous involvement of the hand compromises future investigation into the optimal treatment for such an occurrence. This case underscores the importance of early referral of hand masses to a hand surgeon specialist. It is important to note that this is a case that was done over a decade ago, for which the treatment has and still is advancing. The most common peripheral T‐cell lymphoma remains a diagnosis by exclusion. The current approach includes a PET/CT to stage tumors in their workup, but their treatment remains individualized.^32^, ^33^

## 5 ETHICS STATEMENT

Informed consent was obtained from all individual participants involved in the study.

## CONFLICT OF INTEREST

None declared.

## AUTHOR CONTRIBUTIONS

ML: conceived and designed this patient study, was the principal physician who cared for the patient, and performed the final review; BL: acquired, analyzed, and interpreted the data; JH and MY: prepared, edited, and revised this manuscript for publication.

## Data Availability

The data that support the findings of this study are available on request from Dr Morgan Lorio. The data are not publicly available due to privacy or ethical restrictions.
